# Development and External Validation of a Novel 12-Gene Signature for Prediction of Overall Survival in Muscle-Invasive Bladder Cancer

**DOI:** 10.3389/fonc.2019.00856

**Published:** 2019-09-06

**Authors:** MierXiati Abudurexiti, Huyang Xie, Zhongwei Jia, Yiping Zhu, Yao Zhu, Guohai Shi, Hailiang Zhang, Bo Dai, Fangning Wan, Yijun Shen, Dingwei Ye

**Affiliations:** ^1^Department of Urology, Fudan University Shanghai Cancer Center, Shanghai, China; ^2^Department of Oncology, Shanghai Medical College, Fudan University, Shanghai, China; ^3^Department of Urology, Affiliated Hospital of Nantong University, Nantong, China; ^4^Department of Medical Oncology, Clinical Medical College of Yangzhou University, Northern Jiangsu People's Hospital, Yangzhou, China

**Keywords:** muscle-invasive bladder cancer, gene signature, overall survival, prognosis, TCGA

## Abstract

**Purpose:** We aimed to develop and validate a novel gene signature from published data and improve the prediction of survival in muscle-invasive bladder cancer (MIBC).

**Methods:** We searched the published gene signatures associated with the overall survival (OS) of MIBC and compiled all 274 genes to develop a novel gene signature. RNAseq data of TCGA (the Cancer Genome Atlas) bladder cohort were downloaded. All genes were included in a univariate Cox hazard ratio model. We then used a reduced multivariate Cox regression model, which included only genes achieving *P* < 0.05 in the univariate model. A total of 172 patients at Fudan University Shanghai Cancer Center (FUSCC) and 61 patients from GEO datasets were used as an external validation set.

**Results:** A total of 327 patients in the TCGA cohort were enrolled. We identified 274 genes from eight published papers on the OS of MIBC. Using the TCGA database, we identified 12 genes that correlated with OS (*P* < 0.05 in both univariate and multivariate analyses). By integrating these genes with the RT-qPCR data in our validation dataset and GEO datasets, we confirmed that the power for predicting OS of the 12-gene panel (AUC of 0.741 and 0.727, respectively) was higher than just clinical data (including gender, age, T stage, grade, and N stage) alone in the TCGA and FUSCC cohort (AUC of 0.667 and 0.631, respectively). Additionally, upon combining the clinical data and 12-gene panel together, the AUC increased to 0.768, 0.757, and 0.88 in the TCGA, FUSCC and GSE13507 cohorts, respectively.

**Conclusions:** Applying published gene signatures and TCGA data, we successfully built and externally validated a novel 12-gene signature for the survival of MIBC.

**Brief Explanation:**

We systemically reviewed all published prognostic gene signatures of muscle-invasive bladder cancer (MIBC) and integrated the genes in the TCGA MIBC cohort. This new gene panel was validated in a newly established MIBC cohort in GEO and FUSCC. This method can help update the previous established panels in a new way.

## Introduction

Bladder cancer is the fourth most common cancer, with an incidence of ~7% among all male malignancies, and the eighth most common cause of mortality in men (1). In 2015, a total of 80,500 new bladder cancer cases were expected in China, with 32,900 estimated cancer-related deaths (2). Urothelial carcinoma is the dominant histological subtype of bladder cancer, except in certain parts of Africa and the Middle East (3). Despite the considerable progress in the treatment of bladder cancer, the prognosis of patients with muscle-invasive bladder cancer (MIBC) remains poor, which is partly attributable to the heterogeneity of disease characteristics (4). This indicates the need for an accurate prognostic assessment after radical cystectomy that is essential for treatment decision-making, patient counseling, and most importantly for defining the indication of adjuvant chemotherapy (5).

The American Joint Committee on Cancer (AJCC) TNM staging system, which has been appropriately validated, is the most widely used prognostic model to predict outcome in patients treated with radical cystectomy (6). Although these staging systems provide useful estimates of clinical outcome, their major limitation is the difficulty of incorporating novel clinical information, such as molecular markers or more complex bioinformatics. Furthermore, current staging systems have been shown to be less accurate than some prediction models that incorporate several sets of clinical data in the era of personalized medicine (7). A recently reported, comprehensive molecular analysis of urothelial bladder cancer from the Cancer Genome Atlas (TCGA) (http://cancergenome.nih.gov/) has provided novel insights into molecular subgroups and potential therapeutic targets for this disease (8). A few studies on gene signatures associated with tumor characteristics and outcomes for MIBC had already been reported before the release of the TCGA database. We searched the PubMed database and found eight papers that summarize the gene signature regarding the prediction of overall survival (OS). However, as indicated in the paper by Riester et al. (9), the performance of these eight gene signatures regarding the OS of MIBC was not so robust, as most of their C-indexes were <0.70. Additionally, as the management of MIBC and chemotherapy has changed in recent decades, all the gene signatures need to be updated. Therefore, we planned a study to integrate all of the published genes with TCGA RNAseq data to develop a novel gene panel and to validate the panel in our own cohort by qRT-PCR.

We established a novel 12-gene signature using TCGA data that was well-validated in our cohort and shown to be superior to TNM staging. This signature improved the prediction of survival of MIBC patients when combined with conventional clinical data including gender, age, tumor T and N stages, and tumor grade. Our study has refined the gene signature of MIBC integrated with the RNAseq data of TCGA. These results might reveal new therapeutic targets for bladder cancer and may be helpful during consultations with patients to predict prognosis.

## Patients and Methods

### Selection of Published Studies

**Figure 1:**
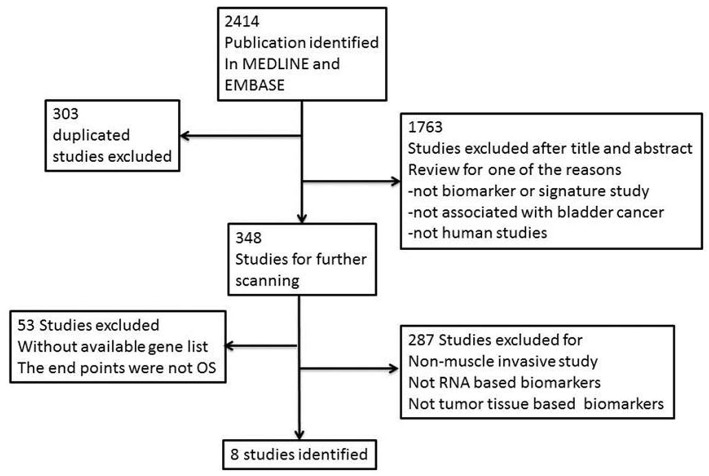
Flow chart of the selection of gene signature studies predicting the OS of MIBC.

We searched EMBASE (www.embase.com) and MEDLINE (www.ncbi.nlm.nih.gov/pubmed) from their inception to December 2017 and systematically identified gene signature studies predicting the OS of MIBC. No language restrictions were applied. The search terms used were as follows: (“bladder cancer” OR “bladder neoplasm” OR “bladder tumor” OR “bladder urothelial carcinoma”) AND (“gene signature” OR “gene profile” OR “gene model” OR “molecular profile” OR “genomic profile” OR “gene expression”). Irrelevant studies were identified and excluded by scanning their titles and abstracts. The full text of the remaining articles was carefully reviewed to determine whether the articles contained information on the topic of interest. We also scanned the cited references of the retrieved articles and reviews to identify any additional relevant studies. Finally, we retrieved all of the gene panels relevant to MIBC and OS (Figure 1).

### Patient Cohorts

Level 3 TCGA RNAseq data from bladder urothelial carcinoma (BLCA) samples were obtained from the TCGA data portal (https://genome-cancer.ucsc.edu/proj/site/hgHeatmap/). Tumor transcriptomic profiles of 20,534 genes were measured in 436 primary bladder cancer patients. Only the 327 patients with intact clinical information, especially follow-up data, were included in this study. The clinical information was retrieved from the “Clinical Biotab” section of the data matrix based on the Biospecimen Core Resource (BCR) identification numbers of the patients. Extended demographic parameters for these patients, characterized by TCGA consortium, are shown in Table 1.

**Table 1:** Clinical characteristics of bladder cancer patients in each cohort.

**Variables**	**TCGA cohort**		**GEO cohort**		**FUSCC cohort**	
	***N* = 327**	**%**	***N* = 61**	**%**	***N* = 172**	**%**
Age, median (range)	69	38–90	66	38–87	63	31–87
**Gender**						
Male	239	73.09	48	78.69	147	85.47
Female	88	26.91	13	21.31	25	14.53
**Grade**						
High	313	95.72	42	68.85	163	94.77
Low	12	3.67	19	31.15	7	4.07
Gx	2	0.61			2	1.16
**pT**						
T0	2	0.61			0	0
T1	2	0.61			5	2.91
T2	95	29.05	31	50.82	63	36.63
T3	154	47.09	19	31.15	41	23.84
T4	49	14.98	11	18.03	29	16.86
Tx	25	7.65			34	19.77
**N**						
N0	190	58.1	46	75.41	106	61.63
N1	35	10.7	8	13.11	12	6.98
N2	64	19.57	6	9.84	19	11.05
N3	7	2.14			0	0
Nx	31	9.48	1	1.64	35	20.35
**M**						
M0	155	47.4	55	90.16	155	90.12
M1	7	2.14	6	9.84	7	4.07
Mx	165	50.46			10	5.81
**Stage**						
I	2	0.61			5	2.91
II	102	31.19	26	42.62	54	31.4
III	108	33.03	13	21.31	37	21.51
IV	111	33.94	6	9.84	52	30.23
X	4	1.22	16	26.23	24	13.95

*TCGA, the cancer genome atlas; FUSCC, Fudan University Shanghai Cancer Center*.

The Fudan University Shanghai Cancer Center (FUSCC) validation cohort consisted of 172 patients with urothelial bladder cancer that was histologically confirmed by an experienced pathologist and treated by radical cystectomy without any pretreatment. These patients were consecutively enrolled from 2008 to 2015 (shown in Table 1). Once resected, tumor tissues were frozen and stored at −80°C. Written informed consent was obtained from all participants of this study. The study protocol was approved by the institutional review board of FUSCC and was carried out in accordance with the approved guidelines (approval ID: 050432-4-1805C).

The GEO dataset GSE13507 was downloaded from the website: www.ncbi.nlm.nih.gov/geo. RNA expression data and metadata of 61 patients were used for external validation of the gene signature. OS data were used for prognosis prediction.

### RNA Preparation, cDNA Synthesis, and qRT-PCR Validation

Total RNA from frozen tissue specimens was extracted using TRIzol reagent (Invitrogen, Carlsbad, CA, USA), in accordance with the manufacturer's instructions. RNA quantity and quality were determined using a NanoDrop ND-1000 Spectrophotometer (NanoDrop Technologies, Wilmington, DE, USA). mRNA levels were measured using a RevertAid First Strand cDNA Synthesis Kit (K1622; Thermo Fisher Scientific, Waltham, MA, USA) and qRT-PCR Kit (Invitrogen, Carlsbad, CA, USA). *ACTB* (β-actin) served as an endogenous control. Primer sets are listed in Supplementary Table 1. qRT-PCR was performed on the Applied Biosystems 7,900 Real-Time PCR system using SYBR Green dye (Applied Biosystems, Foster City, CA, USA), as described by the manufacturer. All determinations were performed in triplicate and in at least three independent experiments. The mean Ct value of each gene minus the mean Ct value of *ACTB* was calculated as ΔCt. The –ΔCt value of each gene was applied for binary logistic regression and model construction. The details of this experiment are shown in our previous paper (10).

### Statistical Analysis

All of the statistical analyses, including gene selection, classification model construction, and independent testing, were performed with R software and packages from the RMS and Bioconductor project (11, 12). For the data obtained by qRT-PCR, univariate and multivariate Cox regression models were used for the selection of genes for the predictive gene signature. All significance tests were two-sided, and a *P* < 0.05 was considered significant. Area under the ROC curve (AUC) was used as an accuracy index to identify the best combination of multiple markers.

## Results

### Acquiring Gene Signatures Associated With the OS of MIBC From Literature Analysis

Two reviewers (H.X. and F.W.) conducted the literature search independently. This resulted in the identification of eight studies (9, 13–19) that met our requirements; all genes included in these studies are featured in our candidate signature gene list after de-replication. All eight studies and the 274 genes are shown in Supplementary Tables 2, 3, respectively.

### Gene List Discovery Using TCGA Database

In the TCGA cohort, we included 239 male and 88 female patients with a median age of 69 years, ranging from 38 to 90 years. Two-thirds of patients were AJCC stage III and IV patients. Since the gene names in the gene models from the different reports were not consistent, we unified the IDs of all 274 genes into their official gene names to find the corresponding genes in the full TCGA gene list. OS data were retrieved from the TCGA cohort and univariate Cox regression was performed to identify the prognostic value of the 274 genes (Supplementary Table 4). In the univariate analysis, 70 genes reached significance at *P* < 0.05. We then performed a reduced model of multivariate Cox regression in the TCGA cohort. The results showed that 12 genes (*ATIC, C6orf62, CPA4, CYFIP2, EGFR, EHBP1, GRK3, MARCH7, QPRT, SARDH, SUZ12*, and *YIF1A*) were factors independently associated with OS (Tables 2, 3).

**Table 2:** Multivariate Cox hazard ratio regression model of integrated gene list in TCGA BLCA cohort.

**Gene IDs**	**OR**	**95%CI**	***P-*value**
ARFGEF1	0.953	(0.441–2.061)	0.902
ARID4B	0.516	(0.246–1.081)	0.080
ATIC	1.698	(1.065–2.708)	**0.026**
BIRC5	1.360	(0.902–2.050)	0.142
C15orf53	0.852	(0.549–1.323)	0.476
C6orf62	0.346	(0.201–0.595)	**0.000**
CALR	1.052	(0.554–1.998)	0.878
CATSPERG	0.868	(0.707–1.066)	0.176
CBX7	0.908	(0.657–1.254)	0.557
CDA	0.857	(0.703–1.044)	0.125
CHD3	1.257	(0.734–2.155)	0.405
COL5A1	1.258	(0.924–1.713)	0.145
CORO1C	1.205	(0.635–2.289)	0.568
CPA4	0.881	(0.785–0.988)	**0.031**
CYFIP2	1.279	(1.011–1.618)	**0.041**
DNASE2B	0.826	(0.627–1.087)	0.173
DPP4	0.987	(0.845–1.153)	0.873
EGFR	1.183	(1.007–1.390)	**0.041**
EHBP1	2.637	(1.575–4.414)	**0.000**
EHF	1.044	(0.869–1.255)	0.645
ENDOD1	1.126	(0.783–1.620)	0.522
ERBB3	1.067	(0.805–1.415)	0.650
ERC1	1.264	(0.771–2.074)	0.353
ESR2	1.043	(0.788–1.380)	0.771
ESYT1	1.367	(0.799–2.388)	0.254
FADD	1.369	(0.956–1.961)	0.086
FN1	0.912	(0.688–1.208)	0.520
FUCA1	1.402	(0.994–1.977)	0.054
FXYD3	0.854	(0.693–1.053)	0.139
GPC3	0.944	(0.826–1.079)	0.402
GRK3	0.752	(0.591–0.957)	**0.021**
HSD17B1	1.122	(0.954–1.318)	0.165
LGALS1	1.055	(0.774–1.438)	0.736
LIMCH1	0.839	(0.681–1.035)	0.101
MAP2K1	1.089	(0.616–1.926)	0.770
MARCH7	0.412	(0.217–0.784)	**0.007**
MECOM	0.920	(0.676–1.250)	0.593
METTL21EP	0.902	(0.650–1.254)	0.540
MMP14	0.988	(0.663–1.472)	0.953
MMP16	0.965	(0.782–1.191)	0.739
MPRIP	1.045	(0.657–1.661)	0.853
NCAPG2	0.688	(0.444–1.065)	0.094
NCLN	1.154	(0.608–2.191)	0.661
NOL12	0.928	(0.552–1.648)	0.798
NOTCH3	1.154	(0.843–1.579)	0.371
PCMTD2	1.564	(0.941–2.600)	0.085
PITX1	1.041	(0.865–1.253)	0.669
PPAPDC1B	0.898	(0.597–1.351)	0.607
PTBP2	1.043	(0.645–1.688)	0.863
PTPN18	1.392	(0.959–2.021)	0.082
QPRT	1.182	(1.003–1.393)	**0.046**
RAD1	0.725	(0.421–1.249)	0.246
RRBP1	1.037	(0.591–1.821)	0.900
RSU1	0.961	(0.593–1.555)	0.870
SARDH	0.732	(0.593–0.904)	**0.004**
SFRS18	1.506	(0.844–2.690)	0.166
SHOX2	1.143	(0.961–1.361)	0.132
SLC16A1	0.968	(0.776–1.206)	0.769
SSRP1	0.854	(0.435–1.675)	0.646
STRAP	1.562	(0.839–2.907)	0.160
SUZ12	1.792	(1.030–1.361)	**0.039**
TBXA2R	0.961	(0.679–1.361)	0.823
TCF7L1	1.018	(0.818–1.266)	0.876
TNFAIP6	0.925	(0.702–1.219)	0.580
TOX3	0.999	(0.895–1.114)	0.978
TRAFD1	0.892	(0.557–1.429)	0.635
VCPIP1	0.996	(0.402–2.469)	0.994
YIF1A	2.197	(1.148–4.207)	**0.018**
ZBTB7B	1.143	(0.732–1.784)	0.558
ZCCHC7	0.695	(0.415–1.164)	0.167

*Parameters that were significant (p < 0.05) in univariate cox regression model entered the multivariate model. Backward Cox regression procedure was used to build the multivariate model; P < 0.05 were indicated as bold type.

**Table 3:** Gene IDs of 12-gene panel.

**Official gene symbol**	**Full name**	**UniGene**
C6orf62	Chromosome 6 open reading frame 62	Hs.744857
YIF1A	Yip1 interacting factor homolog A	Hs.446445
ADRBK2	Adrenergic, beta, receptor kinase 2	Hs.657494
CYFIP2	Cytoplasmic FMR1 interacting protein 2	Hs.519702
ATIC	5-aminoimidazole-4-carboxamide ribonucleotide formyltransferase/IMP cyclohydrolase	Hs.90280
QPRT	Quinolinate phosphoribosyltransferase	Hs.513484
EHBP1	EH domain binding protein 1	Hs.271667
MARCH7	Membrane-associated ring finger (C3HC4) 7, E3 ubiquitin protein ligase	Hs.529272
CPA4	Carboxypeptidase A4	Hs.93764
SUZ12	SUZ12 polycomb repressive complex 2 subunit	Hs.462732
EGFR	Epidermal growth factor receptor	Hs.488293
SARDH	Sarcosine dehydrogenase	Hs.198003

### Validation of the Integrated Gene Signature in the FUSCC Cohort

The FUSCC cohort included 147 male and 25 female patients who underwent cystectomy. The patient age ranged from 31 to 87 years old, with a median of 63 years. We used qRT-PCR to validate all 12 genes in the FUSCC cohort using fresh frozen tissues obtained from cystectomy. The ΔCt value of each gene was normalized by the β-actin Ct value. All 12 genes were significant in the univariate model (all *P* < 0.05, Table 4). In the multivariate Cox regression model, EHBP1 and SARDH were independent prognostic factors.

**Table 4:** Cox hazard ratio analysis of 12-gene signature and OS in FUSCC cohort.

**Gene name**	**Univariate**			**Multivariate**		
	**HR**	**95%CI**	***P***	**HR**	**95%CI**	***P***
ADRBK2	0.704	0.515–0.961	**0.027**	0.727	0.499–1.061	0.100
ATIC	1.680	1.224–2.305	**0.001**	1.269	0.775–2.079	0.346
CYFIP2	1.658	1.198–2.294	**0.002**	1.083	0.635–1.849	0.770
C6orf62	0.621	0.441–0.874	**0.006**	0.807	0.543–1.199	0.290
CPA4	0.664	0.468–0.942	**0.022**	0.736	0.510–1.060	0.101
EHBP1	1.788	1.276–2.505	**0.001**	1.644	1.160–2.330	**0.006**
EGFR	1.720	1.241–2.383	**0.001**	1.202	0.746–1.938	0.452
MARCH7	0.636	0.474–0.853	**0.003**	0.830	0.579–1.194	0.318
SARDH	0.510	0.339–0.766	**0.001**	0.390	0.244–0.623	**0.000**
SUZ12	1.508	1.046–2.174	**0.028**	1.075	0.739–1.578	0.715
QPRT	2.019	1.427–2.856	**0.000**	1.501	1.003–2.247	0.050
YIF1A	1.972	1.400–2.778	**0.000**	1.624	1.061–2.488	0.027

*qRT-PCR were normalized to β-actin. P < 0.05 were indicated as bold type*.

### Integrated Gene Signature and Validation Using GEO Database

RNA expression data and metadata of 61 MIBC patients from GSE13507 were used for external validation of the gene signature. This cohort included 48 male patients and 13 female patients; the median age was 66 years old with a range from 38 to 87 years. All 12 genes were significant in univariate analysis. C6orf62 and ATIC were independent prognosis factors in the multivariate Cox regression model.

### The 12-gene Signature in MIBC Improved the Predictive Value of the Clinical Model

To further assess the prognostic power of the 12-gene signature, we compared this model with a clinical model including gender, age, T stage, tumor grade, and N stage. We used “rms” package in R project to calculate the C-index values of the multivariate cox regression models. The results are shown in Table 5. In TCGA and FUSCC, the 12-gene signature was more accurate than the clinical model with a higher C-index. We then enrolled all the clinical and gene parameters together in a multivariate cox regression model for a combining model. The C-index reached 0.768, 0.757, and 0.88 in the TCGA, FUSCC and GSE13507 cohort, respectively. These results are shown in Table 5.

**Table 5:** C-indexes of Clinical and 12-gene panel prognostic model.

	**TCGA cohort**	**FUSCC cohort**	**GEO cohort**
Clinical data	0.667	0.631	0.772
12-gene panel	0.741	0.727	0.770
Combined model	0.768	0.757	0.880

## Discussion

Once diagnosed, the survival of MIBC patients can range from 1 week to a few years. The disease progression is dependent on risk factors such as tobacco smoking history, exposure to chemicals, radiotherapy, chronic urinary infection, gender, and genetic differences. These clinical criteria may not reflect the entire biology of the disease. In this study, we investigated the efficacy of the 12-gene panel to predict the survival of MIBC patients. Although, previous studies have already developed several gene signatures for predicting OS of MIBC, the management of bladder cancer has improved over decades and the models need to be updated as well. An effective gene signature will improve patient counseling after cystectomy and can better identify candidates who need more aggressive management.

In our study, we performed a meta-analysis to systematically review the literature on gene models and attempted to integrate them together with a relatively newly established public cohort. The updated and integrated novel model should be more applicable to recently treated patients. This approach is relatively novel and has not yet been widely used. In this pilot study, gene expression data from the public TCGA cohort of patients with MIBC were analyzed and external validations were performed using the cohort at our center and GEO datasets. Additionally, we randomly selected 5 genes from the 274 genes, and found the predictive superiority of our 12-gene panel (Supplementary Table 5).

**Figure 2:**
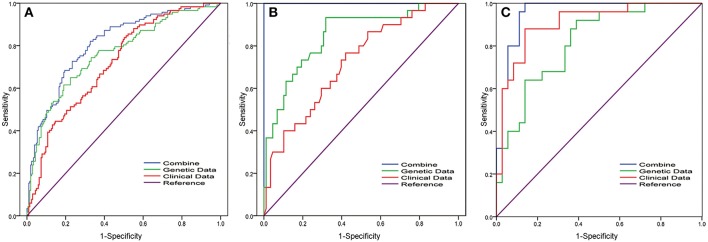
Receiver operating characteristic (ROC) curve analysis of the 12-gene signature in MIBC patients for predicting 5-years overall survival. To compare the prognostic value of the 12-gene signature, we analyzed the ROC curves of the 12-gene signature of 5-years OS in different datasets. ROC plots for the 12-gene signature predicting 5-years OS in the **(A)** TCGA cohort and **(B)** FUSCC cohort and **(C)** GSE13507 dataset. Clinical parameters include gender, age, T stage, grade, and N stage. Combining model was obtained by multivariate regression analysis for the combination of clinical parameter and 12-gene signature.

Leliveld et al. reported that pathological TNM stage and age were independent prognosis factors for patients with MIBC who underwent radical cystectomy (20). Jin S et al. analyzed lymph node-associated variables [pathological N stage (pN), lymph node ratio (LNR) and log odds (LODDDS)] in the patients with MIBC and found c-index that predicted the survival was 0.6769 (pN), 0.6794 (pN+ LNR), and 0.6855 (pN+LNR+LODDDS) (21). Mitra et al. established clinical and genomic classifiers and the c-index was up to 0.73 (18). However, the c-index of the combination model in our study was 0.88. Thus, our update improved the survival prediction. Compared with conventional clinical data, the genomic-clinicopathologic combination in our study had higher clinical benefit by decision curve analysis (Figure 2). These findings are particularly striking given the relative homogeneity of the population analyzed, as our cohort, in which all patients undergone the most aggressive surgical therapy, was strongly selected for being at high risk of death from disease.

Among the members of the novel gene panel, *C6orf62* may participate in suppressing proliferation and inducing differentiation through regulating the cell cycle (22). *YIF1A* is involved in the pathway of the transport of proteins to the Golgi and their subsequent modification, as well as the unfolded protein response. Discrete sites in Yif1A that are necessary for the regulation of endoplasmic reticulum structure have also been identified (23). Moreover, in samples from clinical squamous cell cancer, six genes (*GAL, GSTP1, MRPL11, MRPL21, SF3B2, and YIF1A*) at 11q13.1–13.3 and one gene (*GALR1*) at 18q23 showed significant differences in expression between normal and tumor samples (24).

Another member of the gene panel is *ADRBK2*, which encodes the β-adrenergic receptor kinase, a direct target of CREB activation that regulates the neuroendocrine differentiation of prostate cancer cells (25). This kinase is essential for cell metastasis, promotes prostate tumor progression (26), and regulates breast cancer migration, invasion, and metastasis (27). Another gene panel member is *CYFIP2*, which is involved in T-cell adhesion and p53/TP53-dependent induction of apoptosis. IMP-1 displays cross-talk with K-Ras and modulates colon cancer cell survival through this novel proapoptotic protein (28).

Among the other members of the gene panel, *ATIC* promotes insulin receptor/INSR autophosphorylation and is involved in INSR internalization (29). The small-molecule inhibitor of ATIC has been shown to suppress the proliferation of breast cancer cells (30). *QPRT*, which encodes a key enzyme in the catabolism of quinolinate, an intermediate in the tryptophan-nicotinamide adenine dinucleotide pathway, is a potential marker for follicular thyroid carcinoma including the minimally invasive variant (31). *EHBP1* encodes a protein that may play a role in endocytic trafficking. The single nucleotide polymorphism rs721048(A>G) in *EHBP1* is associated with an aggressive form of prostate cancer (32). *EHBP1* is also essential for the anti-invasive effect of atorvastatin in prostate cancer (33).

The panel also includes *MARCH7*, which is a member of the MARCH family of membrane-bound E3 ubiquitin ligases. E3 ubiquitin ligases accept ubiquitin from an E2 ubiquitin-conjugating enzyme in the form of a thioester and then directly transfer the ubiquitin to targeted substrates. *MARCH7* promotes ovarian tumor growth and its expression is correlated with poor prognosis in epithelial ovarian cancer (34, 35). *CPA4* is also included in the panel and may be involved in the histone hyperacetylation pathway. *CPA4* is imprinted and may be a strong candidate gene for the aggressiveness of prostate cancer (36) as well as a promising diagnostic serum biomarker for both pancreatic cancer and non-small cell lung cancer (37, 38) and an adverse prognostic marker for gastric cancer, NSCLC, and colorectal cancer (37, 39, 40).

The gene panel member *SARDH* encodes an enzyme localized to the mitochondrial matrix that catalyzes the oxidative demethylation of sarcosine. *TMEFF2* and *SARDH* cooperate to modulate one-carbon metabolism and the invasion of prostate cancer cells (41). Another gene in this list is *SUZ12*, which is associated with diseases including endometrial stromal sarcoma and endometrial stromal nodules. Among its related pathways are cellular senescence and chromatin organization. *SUZ12* promotes proliferation and metastasis in many cancers, including gastric cancer (42), colorectal cancer (43), ovarian cancer (44), bladder cancer (45, 46), and NSCLC (47).

The gene encoding epidermal growth factor receptor (EGFR) is also included in the gene panel. EGFR is a receptor tyrosine kinase of the ErbB family. Several studies have shown that the EGFR family of RTKs is involved in urothelial carcinoma progression and chemoresistance. Many clinical trials using inhibitors of EGFR family RTKs have also been performed or are underway (48).

Although it is lack of novelty and function work in our study and our results require further investigation of the efficacy of the 12-gene signature panel in patients, this panel could be extremely beneficial to identify patients at elevated risk of death that may require adjuvant therapy.

## Conclusion

By applying published gene signatures and TCGA data, we successfully built and externally validated a novel 12-gene signature for the survival of MIBC. This model was generated by integration and updating of the existing model. The model improved the prediction of disease progression or survival and may help facilitate doctor-patient consultations and eventually benefit patients.

## Data Availability

All datasets generated for this study are included in the manuscript and/or the Supplementary Files.

## Ethics Statement

This study protocol was approved by the Institutional Review Board of FUSCC and was carried out in accordance with the approved guidelines (approval ID: 050432-4-1805C). As the data (TCGA and GEO datasets) are publicly available, no ethical approval is required.

## Author Contributions

FW, YS, and DY had full access to all the data in the study and take responsibility for the integrity of the data and the accuracy of the data analysis, study concept, design, and supervision. MA and HX acquisition of data and drafting of the manuscript. ZJ, YaZ, and YiZ analysis and interpretation of data. GS, HZ, and BD critical revision of the manuscript for important intellectual content. FW and MA statistical analysis. FW and DY obtaining funding. YaZ and FW administrative, technical, or material support.

### Conflict of Interest Statement

The authors declare that the research was conducted in the absence of any commercial or financial relationships that could be construed as a potential conflict of interest.
