# Supplementary material for: Development and External Validation of a Novel 12-Gene Signature for Prediction of Overall Survival in Muscle-Invasive Bladder Cancer
Source: Front Oncol. 2019 Sep 6;9:856. doi: 10.3389/fonc.2019.00856 (PMC6743371; doi:10.3389/fonc.2019.00856)
Supplement: Supplementary file 1 [file Data_Sheet_1.docx]

Supplementary Table 1. Primers for qRT-PCR of 12 genes discovered in TCGA data mining.

| **Genes** | **Forward primer(5'->3')** | **Reverse primer(5'->3')** |
| --- | --- | --- |
| QPRT | GGGCAGCCTTTCTTCGATG | GGAGCCCATACTTCTCCACCA |
| ADRBK2 | GAAGACCGCCTTTGCAGAAGT | GTGTTCTACAGCTTGCTTTGAGA |
| CYFIP2 | CAACGTGGACCTGCTTGAAGA | AGTTTGTGTCAAAGTTAGCCTGG |
| C6orf62 | CGCAAGGACAAGACTGGAATCG | TGAGCTGTTCCTGTGGCAGGTA |
| SUZ12 | AGGCTGACCACGAGCTTTTC | GGTGCTATGAGATTCCGAGTTC |
| YIF1A | ATGGCTTATCACTCGGGCTAC | CCGCTTGTGTCATCGAAGAGG |
| X7.Mar | CTTCCCGAAGCAATACGCAG | TCGTTGTCCGCCTACCTTCA |
| EHBP1 | TGGTTGAGTGTACGAAGAAATGG | ACAACACCACGATAGGGATTTTT |
| CPA4 | AGGTGGATACTGTTCATTGGGG | TTGCTGATCTCGTCTCCATTTC |
| SARDH | GGAGGAGACGGGACTACACA | AGCCTCTTGTACTCGTCCAGG |
| EGFR | AGGCACGAGTAACAAGCTCAC | ATGAGGACATAACCAGCCACC |
| ATIC | GCAGCCAAAAACCACGCTC | CTGGCGTCTAGTCTCCAAGGA |
| beta-actin | CTGGGACGACATGGAGAAAA | AAGGAAGGCTGGAAGAGTGC |

Supplementary table 2. Gene panel derived from eight former studies.

|  | **Signature studies** | **Gene panel** | **End point** | **Sample type** |
| --- | --- | --- | --- | --- |
| 1 | Emmprin and Survivin Predict Response and Survival following Cisplatin-Containing Chemotherapy in Patients with Advanced Bladder Cancer | 2-gene | Chemotherapy response and OS | MIBC |
| 2 | Discovery and validation of novel expression signature for postcystectomy recurrence in high-risk bladder cancer | 15-gene | Postcystectomy Recurrence and OS | High Risk Bladder Cancer |
| 3 | Defining molecular profiles of poor outcome in patients with invasive bladder cancer using oligonucleotide microarrays. | 174-gene | OS | high risk bladder cancer |
| 4 | Development and prospective evaluation of a 20-gene model for molecular nodal staging of bladder cancer | 20-gene | Lymph node metastasis and OS | MIBC |
| 5 | Combination of a novel gene expression signature with a clinical nomogram improves the prediction of survival in high-risk bladder cancer | 20-gene | OS | MIBC |
| 6 | Generation of a concise gene panel for outcome prediction in urinary bladder cancer. | 4-gene | Recurrence and OS | Ta-T4 |
| 7 | Combined gene expression and genomic profiling define two intrinsic molecular subtypes of urothelial carcinoma and gene signatures for molecular grading and outcome. | 32-gene | OS | MIBC |
| 8 | Predictive value of progression-related gene  classifier in primary non-muscle invasive  bladder cancer | 61-gene | PFS and OS | Non-MIBC and MIBC |

Supplementary table 3. Integrated Gene list derived from eight studies.

| **Gene IDs** | **Full Gene Names** |
| --- | --- |
| AAMP | [angio associated migratory cell protein (AAMP)](https://david.ncifcrf.gov/geneReportFull.jsp?rowids=14) |
| ADAM17 | [ADAM metallopeptidase domain 17(ADAM17)](https://david.ncifcrf.gov/geneReportFull.jsp?rowids=6868) |
| ADM2 | [adrenomedullin 2(ADM2)](https://david.ncifcrf.gov/geneReportFull.jsp?rowids=79924) |
| GRK3 | G Protein-Coupled Receptor Kinase 3 |
| AGPAT3 | [1-acylglycerol-3-phosphate O-acyltransferase 3(AGPAT3)](https://david.ncifcrf.gov/geneReportFull.jsp?rowids=56894) |
| ALDH1A1 | [aldehyde dehydrogenase 1 family member A1(ALDH1A1)](https://david.ncifcrf.gov/geneReportFull.jsp?rowids=216) |
| AMDHD2 | [amidohydrolase domain containing 2(AMDHD2)](https://david.ncifcrf.gov/geneReportFull.jsp?rowids=51005) |
| AP5B1 | [adaptor related protein complex 5 beta 1 subunit(AP5B1)](https://david.ncifcrf.gov/geneReportFull.jsp?rowids=91056) |
| APOBEC3B | [apolipoprotein B mRNA editing enzyme catalytic subunit 3B(APOBEC3B)](https://david.ncifcrf.gov/geneReportFull.jsp?rowids=9582) |
| ARFGEF1 | [ADP ribosylation factor guanine nucleotide exchange factor 1(ARFGEF1)](https://david.ncifcrf.gov/geneReportFull.jsp?rowids=10565) |
| ARID3A | [AT-rich interaction domain 3A(ARID3A)](https://david.ncifcrf.gov/geneReportFull.jsp?rowids=1820) |
| ARID4B | [AT-rich interaction domain 4B(ARID4B)](https://david.ncifcrf.gov/geneReportFull.jsp?rowids=51742) |
| ARL4A | [ADP ribosylation factor like GTPase 4A(ARL4A)](https://david.ncifcrf.gov/geneReportFull.jsp?rowids=10124) |
| ASB12 | [ankyrin repeat and SOCS box containing 12(ASB12)](https://david.ncifcrf.gov/geneReportFull.jsp?rowids=142689) |
| ASXL1 | [additional sex combs like 1, transcriptional regulator(ASXL1)](https://david.ncifcrf.gov/geneReportFull.jsp?rowids=171023) |
| ATAD2 | [ATPase family, AAA domain containing 2(ATAD2)](https://david.ncifcrf.gov/geneReportFull.jsp?rowids=29028) |
| ATF3 | [activating transcription factor 3(ATF3)](https://david.ncifcrf.gov/geneReportFull.jsp?rowids=467) |
| ATIC | [5-aminoimidazole-4-carboxamide ribonucleotide formyltransferase/IMP cyclohydrolase(ATIC)](https://david.ncifcrf.gov/geneReportFull.jsp?rowids=471) |
| ATXN10 | [ataxin 10(ATXN10)](https://david.ncifcrf.gov/geneReportFull.jsp?rowids=25814) |
| AVL9 | [AVL9 cell migration associated(AVL9)](https://david.ncifcrf.gov/geneReportFull.jsp?rowids=23080) |
| BANP | [BTG3 associated nuclear protein(BANP)](https://david.ncifcrf.gov/geneReportFull.jsp?rowids=54971) |
| BIRC5 | [baculoviral IAP repeat containing 5(BIRC5)](https://david.ncifcrf.gov/geneReportFull.jsp?rowids=332) |
| BIRC7 | [baculoviral IAP repeat containing 7(BIRC7)](https://david.ncifcrf.gov/geneReportFull.jsp?rowids=79444) |
| BMP7 | [bone morphogenetic protein 7(BMP7)](https://david.ncifcrf.gov/geneReportFull.jsp?rowids=655) |
| BSPRY | [B-box and SPRY domain containing(BSPRY)](https://david.ncifcrf.gov/geneReportFull.jsp?rowids=54836) |
| BST2 | [bone marrow stromal cell antigen 2(BST2)](https://david.ncifcrf.gov/geneReportFull.jsp?rowids=684) |
| BTBD3 | [BTB domain containing 3(BTBD3)](https://david.ncifcrf.gov/geneReportFull.jsp?rowids=22903) |
| C15orf53 | [chromosome 15 open reading frame 53(C15orf53)](https://david.ncifcrf.gov/geneReportFull.jsp?rowids=400359) |
| C6orf62 | [chromosome 6 open reading frame 62(C6orf62)](https://david.ncifcrf.gov/geneReportFull.jsp?rowids=81688) |
| COQ8A | Coenzyme Q8A |
| CALR | [calreticulin(CALR)](https://david.ncifcrf.gov/geneReportFull.jsp?rowids=811) |
| CAPN1 | [calpain 1(CAPN1)](https://david.ncifcrf.gov/geneReportFull.jsp?rowids=823) |
| CATSPERG | [cation channel sperm associated auxiliary subunit gamma(CATSPERG)](https://david.ncifcrf.gov/geneReportFull.jsp?rowids=57828) |
| CBX7 | [chromobox 7(CBX7)](https://david.ncifcrf.gov/geneReportFull.jsp?rowids=23492) |
| CCDC9 | [coiled-coil domain containing 9(CCDC9)](https://david.ncifcrf.gov/geneReportFull.jsp?rowids=26093) |
| CCL5 | [C-C motif chemokine ligand 5(CCL5)](https://david.ncifcrf.gov/geneReportFull.jsp?rowids=6352) |
| CDA | [cytidine deaminase(CDA)](https://david.ncifcrf.gov/geneReportFull.jsp?rowids=978) |
| CDADC1 | [cytidine and dCMP deaminase domain containing 1(CDADC1)](https://david.ncifcrf.gov/geneReportFull.jsp?rowids=81602) |
| CDAN1 | [codanin 1(CDAN1)](https://david.ncifcrf.gov/geneReportFull.jsp?rowids=146059) |
| CDH2 | [cadherin 2(CDH2)](https://david.ncifcrf.gov/geneReportFull.jsp?rowids=1000) |
| CDH3 | [cadherin 3(CDH3)](https://david.ncifcrf.gov/geneReportFull.jsp?rowids=1001) |
| CHD3 | [chromodomain helicase DNA binding protein 3(CHD3)](https://david.ncifcrf.gov/geneReportFull.jsp?rowids=1107) |
| CHMP2A | [charged multivesicular body protein 2A(CHMP2A)](https://david.ncifcrf.gov/geneReportFull.jsp?rowids=27243) |
| CLCC1 | [chloride channel CLIC like 1(CLCC1)](https://david.ncifcrf.gov/geneReportFull.jsp?rowids=23155) |
| CLDN9 | [claudin 9(CLDN9)](https://david.ncifcrf.gov/geneReportFull.jsp?rowids=9080) |
| CLIC3 | [chloride intracellular channel 3(CLIC3)](https://david.ncifcrf.gov/geneReportFull.jsp?rowids=9022) |
| CNGB1 | [cyclic nucleotide gated channel beta 1(CNGB1)](https://david.ncifcrf.gov/geneReportFull.jsp?rowids=1258) |
| CNTD2 | [cyclin N-terminal domain containing 2(CNTD2)](https://david.ncifcrf.gov/geneReportFull.jsp?rowids=79935) |
| COL5A1 | [collagen type V alpha 1 chain(COL5A1)](https://david.ncifcrf.gov/geneReportFull.jsp?rowids=1289) |
| CORO1C | [coronin 1C(CORO1C)](https://david.ncifcrf.gov/geneReportFull.jsp?rowids=23603) |
| COX20 | [COX20, cytochrome c oxidase assembly factor(COX20)](https://david.ncifcrf.gov/geneReportFull.jsp?rowids=116228) |
| CPA4 | [carboxypeptidase A4(CPA4)](https://david.ncifcrf.gov/geneReportFull.jsp?rowids=51200) |
| CPNE6 | [copine 6(CPNE6)](https://david.ncifcrf.gov/geneReportFull.jsp?rowids=9362) |
| CRAT | [carnitine O-acetyltransferase(CRAT)](https://david.ncifcrf.gov/geneReportFull.jsp?rowids=1384) |
| CRCP | [CGRP receptor component(CRCP)](https://david.ncifcrf.gov/geneReportFull.jsp?rowids=27297) |
| CXCL1 | [C-X-C motif chemokine ligand 1(CXCL1)](https://david.ncifcrf.gov/geneReportFull.jsp?rowids=2919) |
| CXCL3 | [C-X-C motif chemokine ligand 3(CXCL3)](https://david.ncifcrf.gov/geneReportFull.jsp?rowids=2921) |
| CYFIP2 | [cytoplasmic FMR1 interacting protein 2(CYFIP2)](https://david.ncifcrf.gov/geneReportFull.jsp?rowids=26999) |
| DCTN2 | [dynactin subunit 2(DCTN2)](https://david.ncifcrf.gov/geneReportFull.jsp?rowids=10540) |
| DCUN1D4 | [defective in cullin neddylation 1 domain containing 4(DCUN1D4)](https://david.ncifcrf.gov/geneReportFull.jsp?rowids=23142) |
| DDX49 | [DEAD-box helicase 49(DDX49)](https://david.ncifcrf.gov/geneReportFull.jsp?rowids=54555) |
| DDX58 | [DExD/H-box helicase 58(DDX58)](https://david.ncifcrf.gov/geneReportFull.jsp?rowids=23586) |
| DEK | [DEK proto-oncogene(DEK)](https://david.ncifcrf.gov/geneReportFull.jsp?rowids=7913) |
| DGCR2 | [DiGeorge syndrome critical region gene 2(DGCR2)](https://david.ncifcrf.gov/geneReportFull.jsp?rowids=9993) |
| DNAJB6 | [DnaJ heat shock protein family (Hsp40) member B6(DNAJB6)](https://david.ncifcrf.gov/geneReportFull.jsp?rowids=10049) |
| DNASE2B | [deoxyribonuclease 2 beta(DNASE2B)](https://david.ncifcrf.gov/geneReportFull.jsp?rowids=58511) |
| DOCK9 | [dedicator of cytokinesis 9(DOCK9)](https://david.ncifcrf.gov/geneReportFull.jsp?rowids=23348) |
| DPP4 | [dipeptidyl peptidase 4(DPP4)](https://david.ncifcrf.gov/geneReportFull.jsp?rowids=1803) |
| DUSP6 | [dual specificity phosphatase 6(DUSP6)](https://david.ncifcrf.gov/geneReportFull.jsp?rowids=1848) |
| EGFR | [epidermal growth factor receptor(EGFR)](https://david.ncifcrf.gov/geneReportFull.jsp?rowids=1956) |
| EHBP1 | [EH domain binding protein 1(EHBP1)](https://david.ncifcrf.gov/geneReportFull.jsp?rowids=23301) |
| EHF | [ETS homologous factor(EHF)](https://david.ncifcrf.gov/geneReportFull.jsp?rowids=26298) |
| ELFN2 | [extracellular leucine rich repeat and fibronectin type III domain containing 2(ELFN2)](https://david.ncifcrf.gov/geneReportFull.jsp?rowids=114794) |
| EML4 | [echinoderm microtubule associated protein like 4(EML4)](https://david.ncifcrf.gov/geneReportFull.jsp?rowids=27436) |
| ENAH | [enabled homolog (Drosophila)(ENAH)](https://david.ncifcrf.gov/geneReportFull.jsp?rowids=55740) |
| ENDOD1 | [endonuclease domain containing 1(ENDOD1)](https://david.ncifcrf.gov/geneReportFull.jsp?rowids=23052) |
| EPHB3 | [EPH receptor B3(EPHB3)](https://david.ncifcrf.gov/geneReportFull.jsp?rowids=2049) |
| [ERBIN](https://www.genecards.org/cgi-bin/carddisp.pl?gene=ERBIN&keywords=ERBB2IP) | Erbb2 Interacting Protein |
| ERBB3 | [erb-b2 receptor tyrosine kinase 3(ERBB3)](https://david.ncifcrf.gov/geneReportFull.jsp?rowids=2065) |
| ERC1 | [ELKS/RAB6-interacting/CAST family member 1(ERC1)](https://david.ncifcrf.gov/geneReportFull.jsp?rowids=23085) |
| ERLIN2 | [ER lipid raft associated 2(ERLIN2)](https://david.ncifcrf.gov/geneReportFull.jsp?rowids=11160) |
| ESR2 | [estrogen receptor 2(ESR2)](https://david.ncifcrf.gov/geneReportFull.jsp?rowids=2100) |
| ESYT1 | [extended synaptotagmin 1(ESYT1)](https://david.ncifcrf.gov/geneReportFull.jsp?rowids=23344) |
| ETFDH | [electron transfer flavoprotein dehydrogenase(ETFDH)](https://david.ncifcrf.gov/geneReportFull.jsp?rowids=2110) |
| EZH1 | [enhancer of zeste 1 polycomb repressive complex 2 subunit(EZH1)](https://david.ncifcrf.gov/geneReportFull.jsp?rowids=2145) |
| EZH2 | [enhancer of zeste 2 polycomb repressive complex 2 subunit(EZH2)](https://david.ncifcrf.gov/geneReportFull.jsp?rowids=2146) |
| FADD | [Fas associated via death domain(FADD)](https://david.ncifcrf.gov/geneReportFull.jsp?rowids=8772) |
| FAM102B | [family with sequence similarity 102 member B(FAM102B)](https://david.ncifcrf.gov/geneReportFull.jsp?rowids=284611) |
| TMEM255B | Transmembrane Protein 255B |
| FBXO21 | [F-box protein 21(FBXO21)](https://david.ncifcrf.gov/geneReportFull.jsp?rowids=23014) |
| FGFR2 | [fibroblast growth factor receptor 2(FGFR2)](https://david.ncifcrf.gov/geneReportFull.jsp?rowids=2263) |
| FN1 | [fibronectin 1(FN1)](https://david.ncifcrf.gov/geneReportFull.jsp?rowids=2335) |
| FUCA1 | [fucosidase, alpha-L- 1, tissue(FUCA1)](https://david.ncifcrf.gov/geneReportFull.jsp?rowids=2517) |
| FXYD3 | [FXYD domain containing ion transport regulator 3(FXYD3)](https://david.ncifcrf.gov/geneReportFull.jsp?rowids=5349) |
| GABARAPL1 | [GABA type A receptor associated protein like 1(GABARAPL1)](https://david.ncifcrf.gov/geneReportFull.jsp?rowids=23710) |
| GGT1 | [gamma-glutamyltransferase 1(GGT1)](https://david.ncifcrf.gov/geneReportFull.jsp?rowids=2678) |
| GH1 | [growth hormone 1(GH1)](https://david.ncifcrf.gov/geneReportFull.jsp?rowids=2688) |
| GNPTG | [N-acetylglucosamine-1-phosphate transferase gamma subunit(GNPTG)](https://david.ncifcrf.gov/geneReportFull.jsp?rowids=84572) |
| GORASP2 | [golgi reassembly stacking protein 2(GORASP2)](https://david.ncifcrf.gov/geneReportFull.jsp?rowids=26003) |
| GPC3 | [glypican 3(GPC3)](https://david.ncifcrf.gov/geneReportFull.jsp?rowids=2719) |
| GRIN3B | [glutamate ionotropic receptor NMDA type subunit 3B(GRIN3B)](https://david.ncifcrf.gov/geneReportFull.jsp?rowids=116444) |
| GRN | [granulin precursor(GRN)](https://david.ncifcrf.gov/geneReportFull.jsp?rowids=2896) |
| HAGH | [hydroxyacylglutathione hydrolase(HAGH)](https://david.ncifcrf.gov/geneReportFull.jsp?rowids=3029) |
| HLA-G | [major histocompatibility complex, class I, G(HLA-G)](https://david.ncifcrf.gov/geneReportFull.jsp?rowids=3135) |
| HMGB2 | [high mobility group box 2(HMGB2)](https://david.ncifcrf.gov/geneReportFull.jsp?rowids=3148) |
| HN1L | [hematological and neurological expressed 1 like(HN1L)](https://david.ncifcrf.gov/geneReportFull.jsp?rowids=90861) |
| HNRNPA1 | [heterogeneous nuclear ribonucleoprotein A1(HNRNPA1)](https://david.ncifcrf.gov/geneReportFull.jsp?rowids=3178) |
| HRG | [histidine rich glycoprotein(HRG)](https://david.ncifcrf.gov/geneReportFull.jsp?rowids=3273) |
| HSD17B1 | [hydroxysteroid 17-beta dehydrogenase 1(HSD17B1)](https://david.ncifcrf.gov/geneReportFull.jsp?rowids=3292) |
| HSD17B7 | [hydroxysteroid 17-beta dehydrogenase 7(HSD17B7)](https://david.ncifcrf.gov/geneReportFull.jsp?rowids=51478) |
| HSD17B8 | [hydroxysteroid 17-beta dehydrogenase 8(HSD17B8)](https://david.ncifcrf.gov/geneReportFull.jsp?rowids=7923) |
| ICAM1 | [intercellular adhesion molecule 1(ICAM1)](https://david.ncifcrf.gov/geneReportFull.jsp?rowids=3383) |
| IDH3G | [isocitrate dehydrogenase 3 (NAD(+)) gamma(IDH3G)](https://david.ncifcrf.gov/geneReportFull.jsp?rowids=3421) |
| IFI27 | [interferon alpha inducible protein 27(IFI27)](https://david.ncifcrf.gov/geneReportFull.jsp?rowids=3429) |
| IFNA10 | [interferon alpha 10(IFNA10)](https://david.ncifcrf.gov/geneReportFull.jsp?rowids=3446) |
| IGFL2 | [IGF like family member 2(IGFL2)](https://david.ncifcrf.gov/geneReportFull.jsp?rowids=147920) |
| IL1B | [interleukin 1 beta(IL1B)](https://david.ncifcrf.gov/geneReportFull.jsp?rowids=3553) |
| IL6ST | [interleukin 6 signal transducer(IL6ST)](https://david.ncifcrf.gov/geneReportFull.jsp?rowids=3572) |
| CXCL8 | C-X-C Motif Chemokine Ligand 8 |
| IRX2 | [iroquois homeobox 2(IRX2)](https://david.ncifcrf.gov/geneReportFull.jsp?rowids=153572) |
| JUN | [Jun proto-oncogene, AP-1 transcription factor subunit(JUN)](https://david.ncifcrf.gov/geneReportFull.jsp?rowids=3725) |
| JUNB | [JunB proto-oncogene, AP-1 transcription factor subunit(JUNB)](https://david.ncifcrf.gov/geneReportFull.jsp?rowids=3726) |
| KCNC4 | [potassium voltage-gated channel subfamily C member 4(KCNC4)](https://david.ncifcrf.gov/geneReportFull.jsp?rowids=3749) |
| KCNIP1 | [potassium voltage-gated channel interacting protein 1(KCNIP1)](https://david.ncifcrf.gov/geneReportFull.jsp?rowids=30820) |
| KCTD12 | [potassium channel tetramerization domain containing 12(KCTD12)](https://david.ncifcrf.gov/geneReportFull.jsp?rowids=115207) |
| KRT17 | [keratin 17(KRT17)](https://david.ncifcrf.gov/geneReportFull.jsp?rowids=3872) |
| LETMD1 | [LETM1 domain containing 1(LETMD1)](https://david.ncifcrf.gov/geneReportFull.jsp?rowids=25875) |
| LGALS1 | [galectin 1(LGALS1)](https://david.ncifcrf.gov/geneReportFull.jsp?rowids=3956) |
| LHX1 | [LIM homeobox 1(LHX1)](https://david.ncifcrf.gov/geneReportFull.jsp?rowids=3975) |
| LIMCH1 | [LIM and calponin homology domains 1(LIMCH1)](https://david.ncifcrf.gov/geneReportFull.jsp?rowids=22998) |
| LIPG | [lipase G, endothelial type(LIPG)](https://david.ncifcrf.gov/geneReportFull.jsp?rowids=9388) |
| LMO7 | [LIM domain 7(LMO7)](https://david.ncifcrf.gov/geneReportFull.jsp?rowids=4008) |
| LMTK3 | [lemur tyrosine kinase 3(LMTK3)](https://david.ncifcrf.gov/geneReportFull.jsp?rowids=114783) |
| METTL21EP | Methyltransferase Like 21E, Pseudogene |
| LRBA | [LPS responsive beige-like anchor protein(LRBA)](https://david.ncifcrf.gov/geneReportFull.jsp?rowids=987) |
| LRRFIP1 | [LRR binding FLII interacting protein 1(LRRFIP1)](https://david.ncifcrf.gov/geneReportFull.jsp?rowids=9208) |
| MAP2K1 | [mitogen-activated protein kinase kinase 1(MAP2K1)](https://david.ncifcrf.gov/geneReportFull.jsp?rowids=5604) |
| MAP2K6 | [mitogen-activated protein kinase kinase 6(MAP2K6)](https://david.ncifcrf.gov/geneReportFull.jsp?rowids=5608) |
| MAP3K1 | [mitogen-activated protein kinase kinase kinase 1(MAP3K1)](https://david.ncifcrf.gov/geneReportFull.jsp?rowids=4214) |
| MAP4K3 | [mitogen-activated protein kinase kinase kinase kinase 3(MAP4K3)](https://david.ncifcrf.gov/geneReportFull.jsp?rowids=8491) |
| MAP4K4 | [mitogen-activated protein kinase kinase kinase kinase 4(MAP4K4)](https://david.ncifcrf.gov/geneReportFull.jsp?rowids=9448) |
| MARCH7 | [membrane associated ring-CH-type finger 7(MARCH7)](https://david.ncifcrf.gov/geneReportFull.jsp?rowids=64844) |
| MCM7 | [minichromosome maintenance complex component 7(MCM7)](https://david.ncifcrf.gov/geneReportFull.jsp?rowids=4176) |
| MECOM | [MDS1 and EVI1 complex locus(MECOM)](https://david.ncifcrf.gov/geneReportFull.jsp?rowids=2122) |
| MED13L | [mediator complex subunit 13 like(MED13L)](https://david.ncifcrf.gov/geneReportFull.jsp?rowids=23389) |
| MEGF6 | [multiple EGF like domains 6(MEGF6)](https://david.ncifcrf.gov/geneReportFull.jsp?rowids=1953) |
| METTL7A | [methyltransferase like 7A(METTL7A)](https://david.ncifcrf.gov/geneReportFull.jsp?rowids=25840) |
| NIM1K | NIM1 serine/threonine protein kinase |
| MIIP | [migration and invasion inhibitory protein(MIIP)](https://david.ncifcrf.gov/geneReportFull.jsp?rowids=60672) |
| MMP11 | [matrix metallopeptidase 11(MMP11)](https://david.ncifcrf.gov/geneReportFull.jsp?rowids=4320) |
| MMP14 | [matrix metallopeptidase 14(MMP14)](https://david.ncifcrf.gov/geneReportFull.jsp?rowids=4323) |
| MMP16 | [matrix metallopeptidase 16(MMP16)](https://david.ncifcrf.gov/geneReportFull.jsp?rowids=4325) |
| MPRIP | [myosin phosphatase Rho interacting protein(MPRIP)](https://david.ncifcrf.gov/geneReportFull.jsp?rowids=23164) |
| MRPL18 | [mitochondrial ribosomal protein L18(MRPL18)](https://david.ncifcrf.gov/geneReportFull.jsp?rowids=29074) |
| MRPL35 | [mitochondrial ribosomal protein L35(MRPL35)](https://david.ncifcrf.gov/geneReportFull.jsp?rowids=51318) |
| MRPS34 | [mitochondrial ribosomal protein S34(MRPS34)](https://david.ncifcrf.gov/geneReportFull.jsp?rowids=65993) |
| MT1E | [metallothionein 1E(MT1E)](https://david.ncifcrf.gov/geneReportFull.jsp?rowids=4493) |
| MTO1 | [mitochondrial tRNA translation optimization 1(MTO1)](https://david.ncifcrf.gov/geneReportFull.jsp?rowids=25821) |
| MUT | [methylmalonyl-CoA mutase(MUT)](https://david.ncifcrf.gov/geneReportFull.jsp?rowids=4594) |
| MYO1B | [myosin IB(MYO1B)](https://david.ncifcrf.gov/geneReportFull.jsp?rowids=4430) |
| MYO7A | [myosin VIIA(MYO7A)](https://david.ncifcrf.gov/geneReportFull.jsp?rowids=4647) |
| MYO9B | [myosin IXB(MYO9B)](https://david.ncifcrf.gov/geneReportFull.jsp?rowids=4650) |
| NCAPG2 | [non-SMC condensin II complex subunit G2(NCAPG2)](https://david.ncifcrf.gov/geneReportFull.jsp?rowids=54892) |
| NCLN | [nicalin(NCLN)](https://david.ncifcrf.gov/geneReportFull.jsp?rowids=56926) |
| NCOA2 | [nuclear receptor coactivator 2(NCOA2)](https://david.ncifcrf.gov/geneReportFull.jsp?rowids=10499) |
| NEK1 | [NIMA related kinase 1(NEK1)](https://david.ncifcrf.gov/geneReportFull.jsp?rowids=4750) |
| NEK3 | [NIMA related kinase 3(NEK3)](https://david.ncifcrf.gov/geneReportFull.jsp?rowids=4752) |
| NEUROD6 | [neuronal differentiation 6(NEUROD6)](https://david.ncifcrf.gov/geneReportFull.jsp?rowids=63974) |
| NF1 | [neurofibromin 1(NF1)](https://david.ncifcrf.gov/geneReportFull.jsp?rowids=4763) |
| BEX3 | Brain Expressed X-Linked 3 |
| NOC3L | [NOC3 like DNA replication regulator(NOC3L)](https://david.ncifcrf.gov/geneReportFull.jsp?rowids=64318) |
| NOL12 | [nucleolar protein 12(NOL12)](https://david.ncifcrf.gov/geneReportFull.jsp?rowids=79159) |
| NOSIP | [nitric oxide synthase interacting protein(NOSIP)](https://david.ncifcrf.gov/geneReportFull.jsp?rowids=51070) |
| NOTCH3 | [notch 3(NOTCH3)](https://david.ncifcrf.gov/geneReportFull.jsp?rowids=4854) |
| NPEPL1 | [aminopeptidase-like 1(NPEPL1)](https://david.ncifcrf.gov/geneReportFull.jsp?rowids=79716) |
| NRP1 | [neuropilin 1(NRP1)](https://david.ncifcrf.gov/geneReportFull.jsp?rowids=8829) |
| NUDT1 | [nudix hydrolase 1(NUDT1)](https://david.ncifcrf.gov/geneReportFull.jsp?rowids=4521) |
| NUP98 | [nucleoporin 98(NUP98)](https://david.ncifcrf.gov/geneReportFull.jsp?rowids=4928) |
| NXN | [nucleoredoxin(NXN)](https://david.ncifcrf.gov/geneReportFull.jsp?rowids=64359) |
| OAZ3 | [ornithine decarboxylase antizyme 3(OAZ3)](https://david.ncifcrf.gov/geneReportFull.jsp?rowids=51686) |
| OPA1 | [OPA1, mitochondrial dynamin like GTPase(OPA1)](https://david.ncifcrf.gov/geneReportFull.jsp?rowids=4976) |
| OPLAH | [5-oxoprolinase (ATP-hydrolysing)(OPLAH)](https://david.ncifcrf.gov/geneReportFull.jsp?rowids=26873) |
| PBX2 | [PBX homeobox 2(PBX2)](https://david.ncifcrf.gov/geneReportFull.jsp?rowids=5089) |
| PCDHB6 | [protocadherin beta 6(PCDHB6)](https://david.ncifcrf.gov/geneReportFull.jsp?rowids=56130) |
| PCDHGA10 | [protocadherin gamma subfamily A, 10(PCDHGA10)](https://david.ncifcrf.gov/geneReportFull.jsp?rowids=56106) |
| PCM1 | [pericentriolar material 1(PCM1)](https://david.ncifcrf.gov/geneReportFull.jsp?rowids=5108) |
| PCMTD2 | [protein-L-isoaspartate (D-aspartate) O-methyltransferase domain containing 2(PCMTD2)](https://david.ncifcrf.gov/geneReportFull.jsp?rowids=55251) |
| PDGFC | [platelet derived growth factor C(PDGFC)](https://david.ncifcrf.gov/geneReportFull.jsp?rowids=56034) |
| PEA15 | [phosphoprotein enriched in astrocytes 15(PEA15)](https://david.ncifcrf.gov/geneReportFull.jsp?rowids=8682) |
| PFDN6 | [prefoldin subunit 6(PFDN6)](https://david.ncifcrf.gov/geneReportFull.jsp?rowids=10471) |
| PFN1 | [profilin 1(PFN1)](https://david.ncifcrf.gov/geneReportFull.jsp?rowids=5216) |
| PITX1 | [paired like homeodomain 1(PITX1)](https://david.ncifcrf.gov/geneReportFull.jsp?rowids=5307) |
| PLXND1 | [plexin D1(PLXND1)](https://david.ncifcrf.gov/geneReportFull.jsp?rowids=23129) |
| POLM | [DNA polymerase mu(POLM)](https://david.ncifcrf.gov/geneReportFull.jsp?rowids=27434) |
| PLPP5 | Phospholipid Phosphatase 5 |
| PPARD | [peroxisome proliferator activated receptor delta(PPARD)](https://david.ncifcrf.gov/geneReportFull.jsp?rowids=5467) |
| PPP1R12A | [protein phosphatase 1 regulatory subunit 12A(PPP1R12A)](https://david.ncifcrf.gov/geneReportFull.jsp?rowids=4659) |
| PPP1R14C | [protein phosphatase 1 regulatory inhibitor subunit 14C(PPP1R14C)](https://david.ncifcrf.gov/geneReportFull.jsp?rowids=81706) |
| PRAMEF1 | [PRAME family member 1(PRAMEF1)](https://david.ncifcrf.gov/geneReportFull.jsp?rowids=65121) |
| PRDX1 | [peroxiredoxin 1(PRDX1)](https://david.ncifcrf.gov/geneReportFull.jsp?rowids=5052) |
| PRKCA | [protein kinase C alpha(PRKCA)](https://david.ncifcrf.gov/geneReportFull.jsp?rowids=5578) |
| PRKG2 | [protein kinase, cGMP-dependent, type II(PRKG2)](https://david.ncifcrf.gov/geneReportFull.jsp?rowids=5593) |
| PRMT1 | [protein arginine methyltransferase 1(PRMT1)](https://david.ncifcrf.gov/geneReportFull.jsp?rowids=3276) |
| PTBP2 | [polypyrimidine tract binding protein 2(PTBP2)](https://david.ncifcrf.gov/geneReportFull.jsp?rowids=58155) |
| PTHLH | [parathyroid hormone like hormone(PTHLH)](https://david.ncifcrf.gov/geneReportFull.jsp?rowids=5744) |
| PTPN18 | [protein tyrosine phosphatase, non-receptor type 18(PTPN18)](https://david.ncifcrf.gov/geneReportFull.jsp?rowids=26469) |
| QPRT | [quinolinate phosphoribosyltransferase(QPRT)](https://david.ncifcrf.gov/geneReportFull.jsp?rowids=23475) |
| RAB14 | [RAB14, member RAS oncogene family(RAB14)](https://david.ncifcrf.gov/geneReportFull.jsp?rowids=51552) |
| RAB15 | [RAB15, member RAS oncogene family(RAB15)](https://david.ncifcrf.gov/geneReportFull.jsp?rowids=376267) |
| RAD1 | [RAD1 checkpoint DNA exonuclease(RAD1)](https://david.ncifcrf.gov/geneReportFull.jsp?rowids=5810) |
| RBBP7 | [RB binding protein 7, chromatin remodeling factor(RBBP7)](https://david.ncifcrf.gov/geneReportFull.jsp?rowids=5931) |
| RDH13 | [retinol dehydrogenase 13(RDH13)](https://david.ncifcrf.gov/geneReportFull.jsp?rowids=112724) |
| RNASE4 | [ribonuclease A family member 4(RNASE4)](https://david.ncifcrf.gov/geneReportFull.jsp?rowids=6038) |
| RNF130 | [ring finger protein 130(RNF130)](https://david.ncifcrf.gov/geneReportFull.jsp?rowids=55819) |
| RPL15 | [ribosomal protein L15(RPL15)](https://david.ncifcrf.gov/geneReportFull.jsp?rowids=6138) |
| RPLP0 | [ribosomal protein lateral stalk subunit P0(RPLP0)](https://david.ncifcrf.gov/geneReportFull.jsp?rowids=6175) |
| RPS28 | [ribosomal protein S28(RPS28)](https://david.ncifcrf.gov/geneReportFull.jsp?rowids=6234) |
| RRBP1 | [ribosome binding protein 1(RRBP1)](https://david.ncifcrf.gov/geneReportFull.jsp?rowids=6238) |
| RRM2 | [ribonucleotide reductase regulatory subunit M2(RRM2)](https://david.ncifcrf.gov/geneReportFull.jsp?rowids=6241) |
| RSU1 | [Ras suppressor protein 1(RSU1)](https://david.ncifcrf.gov/geneReportFull.jsp?rowids=6251) |
| S100A8 | [S100 calcium binding protein A8(S100A8)](https://david.ncifcrf.gov/geneReportFull.jsp?rowids=6279) |
| S100A9 | [S100 calcium binding protein A9(S100A9)](https://david.ncifcrf.gov/geneReportFull.jsp?rowids=6280) |
| SARDH | [sarcosine dehydrogenase(SARDH)](https://david.ncifcrf.gov/geneReportFull.jsp?rowids=1757) |
| SDHA | [succinate dehydrogenase complex flavoprotein subunit A(SDHA)](https://david.ncifcrf.gov/geneReportFull.jsp?rowids=6389) |
| SEMA7A | [semaphorin 7A (John Milton Hagen blood group)(SEMA7A)](https://david.ncifcrf.gov/geneReportFull.jsp?rowids=8482) |
| SERP1 | [stress associated endoplasmic reticulum protein 1(SERP1)](https://david.ncifcrf.gov/geneReportFull.jsp?rowids=27230) |
| SFN | [stratifin(SFN)](https://david.ncifcrf.gov/geneReportFull.jsp?rowids=2810) |
| PNISR | PNN Interacting Serine And Arginine Rich Protein |
| SHOX2 | [short stature homeobox 2(SHOX2)](https://david.ncifcrf.gov/geneReportFull.jsp?rowids=6474) |
| SLC11A2 | [solute carrier family 11 member 2(SLC11A2)](https://david.ncifcrf.gov/geneReportFull.jsp?rowids=4891) |
| SLC16A1 | [solute carrier family 16 member 1(SLC16A1)](https://david.ncifcrf.gov/geneReportFull.jsp?rowids=6566) |
| SLC1A5 | [solute carrier family 1 member 5(SLC1A5)](https://david.ncifcrf.gov/geneReportFull.jsp?rowids=6510) |
| SLC27A3 | [solute carrier family 27 member 3(SLC27A3)](https://david.ncifcrf.gov/geneReportFull.jsp?rowids=11000) |
| SLC3A2 | [solute carrier family 3 member 2(SLC3A2)](https://david.ncifcrf.gov/geneReportFull.jsp?rowids=6520) |
| SNCA | [synuclein alpha(SNCA)](https://david.ncifcrf.gov/geneReportFull.jsp?rowids=6622) |
| SNRPE | [small nuclear ribonucleoprotein polypeptide E(SNRPE)](https://david.ncifcrf.gov/geneReportFull.jsp?rowids=6635) |
| SRP72 | [signal recognition particle 72(SRP72)](https://david.ncifcrf.gov/geneReportFull.jsp?rowids=6731) |
| SSRP1 | [structure specific recognition protein 1(SSRP1)](https://david.ncifcrf.gov/geneReportFull.jsp?rowids=6749) |
| STAT3 | [signal transducer and activator of transcription 3(STAT3)](https://david.ncifcrf.gov/geneReportFull.jsp?rowids=6774) |
| STRAP | [serine/threonine kinase receptor associated protein(STRAP)](https://david.ncifcrf.gov/geneReportFull.jsp?rowids=11171) |
| SUZ12 | [SUZ12 polycomb repressive complex 2 subunit(SUZ12)](https://david.ncifcrf.gov/geneReportFull.jsp?rowids=23512) |
| SYPL1 | [synaptophysin like 1(SYPL1)](https://david.ncifcrf.gov/geneReportFull.jsp?rowids=6856) |
| TBXA2R | [thromboxane A2 receptor(TBXA2R)](https://david.ncifcrf.gov/geneReportFull.jsp?rowids=6915) |
| TCF12 | [transcription factor 12(TCF12)](https://david.ncifcrf.gov/geneReportFull.jsp?rowids=6938) |
| TCF7L1 | [transcription factor 7 like 1(TCF7L1)](https://david.ncifcrf.gov/geneReportFull.jsp?rowids=83439) |
| TCP1 | [t-complex 1(TCP1)](https://david.ncifcrf.gov/geneReportFull.jsp?rowids=6950) |
| TGFB2 | [transforming growth factor beta 2(TGFB2)](https://david.ncifcrf.gov/geneReportFull.jsp?rowids=7042) |
| TGM2 | [transglutaminase 2(TGM2)](https://david.ncifcrf.gov/geneReportFull.jsp?rowids=7052) |
| TMEM132B | [transmembrane protein 132B(TMEM132B)](https://david.ncifcrf.gov/geneReportFull.jsp?rowids=114795) |
| TMEM191A | [transmembrane protein 191A (pseudogene)(TMEM191A)](https://david.ncifcrf.gov/geneReportFull.jsp?rowids=84222) |
| TMEM63A | [transmembrane protein 63A(TMEM63A)](https://david.ncifcrf.gov/geneReportFull.jsp?rowids=9725) |
| TMEM87A | [transmembrane protein 87A(TMEM87A)](https://david.ncifcrf.gov/geneReportFull.jsp?rowids=25963) |
| TNFAIP6 | [TNF alpha induced protein 6(TNFAIP6)](https://david.ncifcrf.gov/geneReportFull.jsp?rowids=7130) |
| TOPORS | [TOP1 binding arginine/serine rich protein(TOPORS)](https://david.ncifcrf.gov/geneReportFull.jsp?rowids=10210) |
| TOX3 | [TOX high mobility group box family member 3(TOX3)](https://david.ncifcrf.gov/geneReportFull.jsp?rowids=27324) |
| TRAFD1 | [TRAF-type zinc finger domain containing 1(TRAFD1)](https://david.ncifcrf.gov/geneReportFull.jsp?rowids=10906) |
| TROAP | [trophinin associated protein(TROAP)](https://david.ncifcrf.gov/geneReportFull.jsp?rowids=10024) |
| TRPM3 | [transient receptor potential cation channel subfamily M member 3(TRPM3)](https://david.ncifcrf.gov/geneReportFull.jsp?rowids=80036) |
| TSG101 | [tumor susceptibility 101(TSG101)](https://david.ncifcrf.gov/geneReportFull.jsp?rowids=7251) |
| TSPAN5 | [tetraspanin 5(TSPAN5)](https://david.ncifcrf.gov/geneReportFull.jsp?rowids=10098) |
| TTC12 | [tetratricopeptide repeat domain 12(TTC12)](https://david.ncifcrf.gov/geneReportFull.jsp?rowids=54970) |
| TTC38 | [tetratricopeptide repeat domain 38(TTC38)](https://david.ncifcrf.gov/geneReportFull.jsp?rowids=55020) |
| TUB | [tubby bipartite transcription factor(TUB)](https://david.ncifcrf.gov/geneReportFull.jsp?rowids=7275) |
| TULP4 | [tubby like protein 4(TULP4)](https://david.ncifcrf.gov/geneReportFull.jsp?rowids=56995) |
| TXN | [thioredoxin(TXN)](https://david.ncifcrf.gov/geneReportFull.jsp?rowids=7295) |
| UBE2Q2 | [ubiquitin conjugating enzyme E2 Q2(UBE2Q2)](https://david.ncifcrf.gov/geneReportFull.jsp?rowids=92912) |
| UGGT2 | [UDP-glucose glycoprotein glucosyltransferase 2(UGGT2)](https://david.ncifcrf.gov/geneReportFull.jsp?rowids=55757) |
| VCPIP1 | [valosin containing protein interacting protein 1(VCPIP1)](https://david.ncifcrf.gov/geneReportFull.jsp?rowids=80124) |
| WNT6 | [Wnt family member 6(WNT6)](https://david.ncifcrf.gov/geneReportFull.jsp?rowids=7475) |
| YIF1A | [Yip1 interacting factor homolog A, membrane trafficking protein(YIF1A)](https://david.ncifcrf.gov/geneReportFull.jsp?rowids=10897) |
| ZBTB7B | [zinc finger and BTB domain containing 7B(ZBTB7B)](https://david.ncifcrf.gov/geneReportFull.jsp?rowids=51043) |
| ZCCHC7 | [zinc finger CCHC-type containing 7(ZCCHC7)](https://david.ncifcrf.gov/geneReportFull.jsp?rowids=84186) |
| ZMAT5 | [zinc finger matrin-type 5(ZMAT5)](https://david.ncifcrf.gov/geneReportFull.jsp?rowids=55954) |
| ZNF45 | [zinc finger protein 45(ZNF45)](https://david.ncifcrf.gov/geneReportFull.jsp?rowids=7596) |

Supplementary table 4. Univariate Cox Hazard regression of integrated gene list.

| Genes | OR | 95%CI_lower | 95%CI_upper | *P** |
| --- | --- | --- | --- | --- |
| AAMP | 0.827 | 0.543 | 1.260 | 0.377 |
| ADAM17 | 1.157 | 0.948 | 1.413 | 0.152 |
| ADM2 | 1.052 | 0.949 | 1.166 | 0.338 |
| GRK3 | 0.811 | 0.700 | 0.940 | **0.005** |
| AGPAT3 | 0.821 | 0.608 | 1.109 | 0.199 |
| ALDH1A1 | 1.015 | 0.948 | 1.086 | 0.671 |
| AMDHD2 | 1.025 | 0.777 | 1.351 | 0.863 |
| AP5B1 | 0.846 | 0.636 | 1.125 | 0.251 |
| APOBEC3B | 1.004 | 0.913 | 1.104 | 0.932 |
| ARFGEF1 | 0.779 | 0.582 | 1.041 | 0.091 |
| ARID3A | 1.091 | 0.978 | 1.218 | 0.118 |
| ARID4B | 0.668 | 0.468 | 0.952 | **0.026** |
| ARL4A | 1.075 | 0.906 | 1.277 | 0.407 |
| ASB12 | 0.913 | 0.791 | 1.054 | 0.213 |
| ASXL1 | 0.845 | 0.605 | 1.179 | 0.321 |
| ATAD2 | 1.086 | 0.887 | 1.329 | 0.426 |
| ATF3 | 0.970 | 0.872 | 1.079 | 0.576 |
| ATIC | 1.311 | 0.951 | 1.808 | 0.098 |
| ATXN10 | 0.992 | 0.712 | 1.384 | 0.964 |
| AVL9 | 1.215 | 0.914 | 1.616 | 0.180 |
| BANP | 0.900 | 0.594 | 1.364 | 0.619 |
| BIRC5 | 1.233 | 1.018 | 1.495 | **0.032** |
| BIRC7 | 0.930 | 0.835 | 1.036 | 0.189 |
| BMP7 | 0.961 | 0.913 | 1.012 | 0.136 |
| BSPRY | 0.988 | 0.890 | 1.098 | 0.828 |
| BST2 | 0.953 | 0.874 | 1.040 | 0.279 |
| BTBD3 | 0.887 | 0.723 | 1.088 | 0.249 |
| C15orf53 | 0.758 | 0.578 | 0.994 | **0.045** |
| C6orf62 | 0.447 | 0.316 | 0.632 | **0.000** |
| COQ8A | 1.033 | 0.828 | 1.290 | 0.773 |
| CALR | 1.610 | 1.118 | 2.318 | **0.010** |
| CAPN1 | 0.864 | 0.667 | 1.120 | 0.270 |
| CATSPERG | 0.854 | 0.753 | 0.967 | **0.013** |
| CBX7 | 0.791 | 0.665 | 0.941 | **0.008** |
| CCDC9 | 0.836 | 0.567 | 1.233 | 0.366 |
| CCL5 | 0.945 | 0.871 | 1.026 | 0.179 |
| CDA | 1.085 | 0.995 | 1.184 | 0.064 |
| CDADC1 | 0.970 | 0.744 | 1.264 | 0.822 |
| CDAN1 | 0.977 | 0.666 | 1.433 | 0.906 |
| CDH2 | 1.046 | 0.970 | 1.128 | 0.238 |
| CDH3 | 0.947 | 0.874 | 1.026 | 0.181 |
| CHD3 | 0.786 | 0.603 | 1.023 | 0.073 |
| CHMP2A | 0.858 | 0.647 | 1.136 | 0.284 |
| CLCC1 | 1.103 | 0.723 | 1.682 | 0.650 |
| CLDN9 | 1.029 | 0.960 | 1.104 | 0.420 |
| CLIC3 | 1.061 | 0.989 | 1.138 | 0.101 |
| CNGB1 | 1.005 | 0.929 | 1.087 | 0.909 |
| CNTD2 | 1.040 | 0.941 | 1.150 | 0.441 |
| COL5A1 | 1.092 | 0.996 | 1.196 | 0.060 |
| CORO1C | 1.332 | 1.079 | 1.644 | **0.008** |
| COX20 | 0.877 | 0.644 | 1.196 | 0.408 |
| CPA4 | 1.066 | 1.007 | 1.129 | **0.028** |
| CPNE6 | 0.932 | 0.818 | 1.063 | 0.295 |
| CRAT | 1.101 | 0.953 | 1.272 | 0.193 |
| CRCP | 0.855 | 0.567 | 1.289 | 0.455 |
| CXCL1 | 0.977 | 0.915 | 1.044 | 0.496 |
| CXCL3 | 1.019 | 0.942 | 1.103 | 0.638 |
| CYFIP2 | 0.904 | 0.807 | 1.013 | 0.084 |
| DCTN2 | 1.254 | 0.788 | 1.996 | 0.339 |
| DCUN1D4 | 0.882 | 0.632 | 1.230 | 0.458 |
| DDX49 | 1.069 | 0.754 | 1.515 | 0.709 |
| DDX58 | 0.947 | 0.824 | 1.089 | 0.444 |
| DEK | 1.036 | 0.840 | 1.277 | 0.740 |
| DGCR2 | 0.818 | 0.590 | 1.134 | 0.227 |
| DNAJB6 | 1.006 | 0.734 | 1.379 | 0.970 |
| DNASE2B | 0.731 | 0.606 | 0.881 | **0.001** |
| DOCK9 | 1.000 | 0.797 | 1.256 | 0.998 |
| DPP4 | 1.091 | 1.008 | 1.180 | **0.031** |
| DUSP6 | 1.036 | 0.896 | 1.197 | 0.636 |
| EGFR | 1.140 | 1.032 | 1.259 | **0.010** |
| EHBP1 | 1.634 | 1.285 | 2.077 | **0.000** |
| EHF | 0.896 | 0.828 | 0.969 | **0.006** |
| ELFN2 | 0.964 | 0.883 | 1.053 | 0.415 |
| EML4 | 0.933 | 0.686 | 1.270 | 0.659 |
| ENAH | 1.167 | 0.949 | 1.435 | 0.144 |
| ENDOD1 | 1.374 | 1.139 | 1.658 | **0.001** |
| EPHB3 | 1.099 | 0.978 | 1.234 | 0.112 |
| ERBB2IP | 0.826 | 0.643 | 1.061 | 0.135 |
| ERBB3 | 0.890 | 0.813 | 0.975 | **0.012** |
| ERC1 | 1.403 | 1.106 | 1.779 | **0.005** |
| ERLIN2 | 0.875 | 0.705 | 1.085 | 0.223 |
| ESR2 | 0.872 | 0.742 | 1.025 | 0.097 |
| ESYT1 | 1.294 | 0.966 | 1.734 | 0.084 |
| ETFDH | 0.900 | 0.644 | 1.259 | 0.539 |
| EZH1 | 0.935 | 0.693 | 1.262 | 0.660 |
| EZH2 | 1.026 | 0.852 | 1.236 | 0.784 |
| FADD | 1.289 | 1.031 | 1.612 | **0.026** |
| FAM102B | 0.968 | 0.804 | 1.165 | 0.732 |
| TMEM255B | 1.019 | 0.871 | 1.194 | 0.811 |
| FBXO21 | 0.944 | 0.690 | 1.291 | 0.718 |
| FGFR2 | 0.947 | 0.874 | 1.027 | 0.187 |
| FN1 | 1.105 | 1.017 | 1.202 | **0.019** |
| FUCA1 | 0.816 | 0.684 | 0.974 | **0.024** |
| FXYD3 | 0.915 | 0.861 | 0.972 | **0.004** |
| GABARAPL1 | 0.959 | 0.771 | 1.192 | 0.706 |
| GGT1 | 0.958 | 0.856 | 1.074 | 0.464 |
| GH1 | 0.887 | 0.600 | 1.313 | 0.550 |
| GNPTG | 0.887 | 0.657 | 1.199 | 0.436 |
| GORASP2 | 0.907 | 0.569 | 1.446 | 0.682 |
| GPC3 | 0.939 | 0.872 | 1.011 | 0.095 |
| GRIN3B | 1.051 | 0.951 | 1.161 | 0.332 |
| GRN | 1.149 | 0.889 | 1.485 | 0.289 |
| HAGH | 0.800 | 0.578 | 1.107 | 0.178 |
| HLA-G | 0.921 | 0.822 | 1.032 | 0.154 |
| HMGB2 | 0.905 | 0.742 | 1.104 | 0.325 |
| HN1L | 1.120 | 0.779 | 1.610 | 0.541 |
| HNRNPA1 | 0.848 | 0.590 | 1.218 | 0.371 |
| HRG | 0.886 | 0.766 | 1.024 | 0.101 |
| HSD17B1 | 1.120 | 1.013 | 1.239 | **0.028** |
| HSD17B7 | 0.915 | 0.708 | 1.181 | 0.495 |
| HSD17B8 | 1.031 | 0.877 | 1.213 | 0.708 |
| ICAM1 | 1.003 | 0.906 | 1.110 | 0.955 |
| IDH3G | 1.055 | 0.757 | 1.471 | 0.752 |
| IFI27 | 1.000 | 0.928 | 1.077 | 0.998 |
| IFNA10 | 0.145 | 0.002 | 11.229 | 0.384 |
| IGFL2 | 1.040 | 0.978 | 1.107 | 0.211 |
| IL1B | 0.964 | 0.894 | 1.040 | 0.347 |
| IL6ST | 1.024 | 0.932 | 1.125 | 0.621 |
| CXCL8 | 0.994 | 0.926 | 1.068 | 0.876 |
| IRX2 | 0.980 | 0.917 | 1.048 | 0.560 |
| JUN | 1.090 | 0.932 | 1.274 | 0.279 |
| JUNB | 0.866 | 0.720 | 1.041 | 0.125 |
| KCNC4 | 1.119 | 0.967 | 1.294 | 0.131 |
| KCNIP1 | 1.009 | 0.850 | 1.198 | 0.918 |
| KCTD12 | 1.001 | 0.877 | 1.143 | 0.985 |
| KRT17 | 0.989 | 0.931 | 1.050 | 0.712 |
| LETMD1 | 0.797 | 0.569 | 1.115 | 0.185 |
| LGALS1 | 1.182 | 1.043 | 1.339 | **0.009** |
| LHX1 | 1.068 | 0.980 | 1.163 | 0.134 |
| LIMCH1 | 0.876 | 0.798 | 0.962 | **0.006** |
| LIPG | 1.043 | 0.957 | 1.136 | 0.341 |
| LMO7 | 1.013 | 0.873 | 1.176 | 0.864 |
| LMTK3 | 0.951 | 0.867 | 1.043 | 0.287 |
| METTL21EP | 0.758 | 0.595 | 0.966 | **0.025** |
| LRBA | 1.100 | 0.865 | 1.398 | 0.439 |
| LRRFIP1 | 0.788 | 0.594 | 1.047 | 0.101 |
| MAP2K1 | 1.586 | 1.095 | 2.298 | **0.015** |
| MAP2K6 | 1.043 | 0.913 | 1.193 | 0.533 |
| MAP3K1 | 0.996 | 0.813 | 1.220 | 0.971 |
| MAP4K3 | 0.864 | 0.694 | 1.075 | 0.189 |
| MAP4K4 | 1.164 | 0.940 | 1.442 | 0.164 |
| MARCH7 | 0.706 | 0.478 | 1.042 | 0.080 |
| MCM7 | 0.991 | 0.779 | 1.262 | 0.945 |
| MECOM | 0.850 | 0.767 | 0.942 | **0.002** |
| MED13L | 1.016 | 0.798 | 1.294 | 0.896 |
| MEGF6 | 0.924 | 0.836 | 1.020 | 0.118 |
| METTL7A | 0.949 | 0.865 | 1.041 | 0.269 |
| NIM1K | 0.997 | 0.867 | 1.146 | 0.964 |
| MIIP | 0.913 | 0.686 | 1.215 | 0.533 |
| MMP11 | 1.036 | 0.961 | 1.116 | 0.355 |
| MMP14 | 1.197 | 1.008 | 1.422 | **0.040** |
| MMP16 | 1.114 | 0.999 | 1.241 | 0.052 |
| MPRIP | 1.359 | 1.052 | 1.755 | **0.019** |
| MRPL18 | 0.931 | 0.629 | 1.377 | 0.720 |
| MRPL35 | 0.784 | 0.483 | 1.274 | 0.326 |
| MRPS34 | 0.901 | 0.652 | 1.245 | 0.527 |
| MT1E | 1.025 | 0.948 | 1.109 | 0.538 |
| MTO1 | 1.097 | 0.757 | 1.591 | 0.624 |
| MUT | 0.978 | 0.696 | 1.373 | 0.896 |
| MYO1B | 1.017 | 0.887 | 1.165 | 0.812 |
| MYO7A | 0.928 | 0.821 | 1.050 | 0.235 |
| MYO9B | 0.840 | 0.564 | 1.249 | 0.389 |
| NCAPG2 | 1.183 | 0.969 | 1.444 | 0.098 |
| NCLN | 1.531 | 1.084 | 2.163 | **0.016** |
| NCOA2 | 1.016 | 0.907 | 1.139 | 0.782 |
| NEK1 | 0.973 | 0.766 | 1.236 | 0.823 |
| NEK3 | 0.925 | 0.777 | 1.102 | 0.384 |
| NEUROD6 | 1.210 | 0.956 | 1.532 | 0.113 |
| NF1 | 0.972 | 0.745 | 1.267 | 0.833 |
| BEX3 | 1.067 | 0.859 | 1.324 | 0.558 |
| NOC3L | 1.171 | 0.832 | 1.647 | 0.366 |
| NOL12 | 0.699 | 0.498 | 0.982 | **0.039** |
| NOSIP | 0.913 | 0.657 | 1.268 | 0.587 |
| NOTCH3 | 1.191 | 0.982 | 1.445 | 0.075 |
| NPEPL1 | 0.845 | 0.648 | 1.100 | 0.211 |
| NRP1 | 1.083 | 0.941 | 1.246 | 0.266 |
| NUDT1 | 1.146 | 0.909 | 1.446 | 0.248 |
| NUP98 | 1.367 | 0.918 | 2.035 | 0.123 |
| NXN | 1.081 | 0.962 | 1.214 | 0.189 |
| OAZ3 | 1.002 | 0.846 | 1.186 | 0.986 |
| OPA1 | 1.002 | 0.687 | 1.462 | 0.990 |
| OPLAH | 1.015 | 0.895 | 1.151 | 0.818 |
| PBX2 | 1.088 | 0.762 | 1.555 | 0.641 |
| PCDHB6 | 1.004 | 0.929 | 1.085 | 0.926 |
| PCDHGA10 | 0.978 | 0.893 | 1.071 | 0.628 |
| PCM1 | 1.094 | 0.830 | 1.442 | 0.523 |
| PCMTD2 | 0.802 | 0.633 | 1.016 | 0.067 |
| PDGFC | 1.074 | 0.955 | 1.209 | 0.235 |
| PEA15 | 1.167 | 0.898 | 1.516 | 0.247 |
| PFDN6 | 0.829 | 0.621 | 1.106 | 0.202 |
| PFN1 | 1.161 | 0.840 | 1.605 | 0.367 |
| PITX1 | 1.088 | 0.985 | 1.202 | 0.096 |
| PLXND1 | 1.143 | 0.956 | 1.368 | 0.142 |
| POLM | 0.893 | 0.660 | 1.209 | 0.464 |
| PLPP5 | 0.721 | 0.575 | 0.905 | **0.005** |
| PPARD | 0.890 | 0.670 | 1.181 | 0.420 |
| PPP1R12A | 0.908 | 0.676 | 1.220 | 0.522 |
| PPP1R14C | 1.013 | 0.951 | 1.078 | 0.694 |
| PRAMEF1 | 1.053 | 0.712 | 1.557 | 0.796 |
| PRDX1 | 1.232 | 0.936 | 1.621 | 0.137 |
| PRKCA | 0.956 | 0.828 | 1.105 | 0.545 |
| PRKG2 | 0.957 | 0.850 | 1.077 | 0.463 |
| PRMT1 | 1.040 | 0.740 | 1.461 | 0.823 |
| PTBP2 | 0.769 | 0.585 | 1.012 | 0.061 |
| PTHLH | 1.035 | 0.974 | 1.099 | 0.274 |
| PTPN18 | 0.813 | 0.638 | 1.037 | 0.095 |
| QPRT | 1.105 | 1.013 | 1.205 | **0.024** |
| RAB14 | 0.858 | 0.571 | 1.289 | 0.460 |
| RAB15 | 0.924 | 0.834 | 1.024 | 0.130 |
| RAD1 | 0.730 | 0.515 | 1.035 | 0.078 |
| RBBP7 | 0.927 | 0.645 | 1.332 | 0.681 |
| RDH13 | 0.894 | 0.723 | 1.105 | 0.299 |
| RNASE4 | 0.958 | 0.838 | 1.095 | 0.527 |
| RNF130 | 1.093 | 0.903 | 1.322 | 0.362 |
| RPL15 | 0.954 | 0.713 | 1.276 | 0.751 |
| RPLP0 | 1.068 | 0.814 | 1.401 | 0.636 |
| RPS28 | 0.999 | 0.941 | 1.060 | 0.970 |
| RRBP1 | 1.394 | 1.042 | 1.863 | **0.025** |
| RRM2 | 1.128 | 0.953 | 1.335 | 0.160 |
| RSU1 | 1.297 | 0.979 | 1.720 | 0.070 |
| S100A8 | 1.024 | 0.974 | 1.076 | 0.353 |
| S100A9 | 1.017 | 0.959 | 1.077 | 0.575 |
| SARDH | 0.880 | 0.782 | 0.991 | **0.035** |
| SDHA | 1.165 | 0.869 | 1.562 | 0.308 |
| SEMA7A | 1.035 | 0.920 | 1.163 | 0.569 |
| SERP1 | 0.801 | 0.527 | 1.217 | 0.298 |
| SFN | 1.057 | 0.947 | 1.179 | 0.324 |
| PNISR | 0.681 | 0.513 | 0.904 | **0.008** |
| SHOX2 | 1.086 | 0.995 | 1.186 | 0.065 |
| SLC11A2 | 1.090 | 0.824 | 1.444 | 0.546 |
| SLC16A1 | 1.115 | 1.025 | 1.213 | **0.011** |
| SLC1A5 | 1.113 | 0.900 | 1.378 | 0.322 |
| SLC27A3 | 1.060 | 0.846 | 1.327 | 0.614 |
| SLC3A2 | 1.171 | 0.950 | 1.443 | 0.138 |
| SNCA | 0.999 | 0.911 | 1.095 | 0.977 |
| SNRPE | 0.847 | 0.586 | 1.224 | 0.377 |
| SRP72 | 1.117 | 0.717 | 1.739 | 0.625 |
| SSRP1 | 1.799 | 1.291 | 2.508 | **0.001** |
| STAT3 | 1.120 | 0.839 | 1.497 | 0.442 |
| STRAP | 1.675 | 1.180 | 2.377 | **0.004** |
| SUZ12 | 1.305 | 0.954 | 1.787 | 0.096 |
| SYPL1 | 0.878 | 0.686 | 1.125 | 0.303 |
| TBXA2R | 1.150 | 0.985 | 1.344 | 0.078 |
| TCF12 | 1.042 | 0.794 | 1.367 | 0.768 |
| TCF7L1 | 1.112 | 0.996 | 1.242 | 0.059 |
| TCP1 | 1.193 | 0.879 | 1.619 | 0.257 |
| TGFB2 | 1.036 | 0.933 | 1.150 | 0.507 |
| TGM2 | 1.014 | 0.931 | 1.104 | 0.751 |
| TMEM132B | 1.010 | 0.892 | 1.144 | 0.875 |
| TMEM191A | 0.885 | 0.757 | 1.034 | 0.125 |
| TMEM63A | 0.869 | 0.713 | 1.060 | 0.166 |
| TMEM87A | 1.095 | 0.755 | 1.588 | 0.634 |
| TNFAIP6 | 1.090 | 1.000 | 1.187 | **0.050** |
| TOPORS | 0.892 | 0.653 | 1.220 | 0.475 |
| TOX3 | 0.953 | 0.915 | 0.993 | **0.022** |
| TRAFD1 | 0.583 | 0.419 | 0.812 | **0.001** |
| TROAP | 1.002 | 0.844 | 1.189 | 0.985 |
| TRPM3 | 1.077 | 0.944 | 1.229 | 0.271 |
| TSG101 | 1.087 | 0.717 | 1.647 | 0.694 |
| TSPAN5 | 1.089 | 0.966 | 1.229 | 0.163 |
| TTC12 | 0.898 | 0.721 | 1.118 | 0.337 |
| TTC38 | 1.090 | 0.851 | 1.398 | 0.495 |
| TUB | 1.066 | 0.960 | 1.184 | 0.231 |
| TULP4 | 0.981 | 0.700 | 1.375 | 0.911 |
| TXN | 0.962 | 0.781 | 1.185 | 0.715 |
| UBE2Q2 | 0.968 | 0.726 | 1.291 | 0.826 |
| UGGT2 | 0.957 | 0.739 | 1.239 | 0.741 |
| VCPIP1 | 0.676 | 0.479 | 0.955 | **0.026** |
| WNT6 | 1.014 | 0.943 | 1.091 | 0.710 |
| YIF1A | 1.680 | 1.226 | 2.304 | **0.001** |
| ZBTB7B | 0.808 | 0.643 | 1.015 | 0.067 |
| ZCCHC7 | 0.740 | 0.533 | 1.027 | 0.072 |
| ZMAT5 | 0.911 | 0.703 | 1.182 | 0.484 |
| ZNF45 | 0.897 | 0.665 | 1.209 | 0.476 |

*Parameters that were significant(p<0.05) in univariate cox regression model entered the multivariate model.

*P*<0.05 were indicated as bold type

Supplementary table 5. Cox Hazard Ratio analysis of 5 random genes signature and OS in FUSCC Cohort

| Gene Name | Univariate | | | Multivariate | | |
| --- | --- | --- | --- | --- | --- | --- |
|  | HR | 95%CI | P | HR | 95%CI | P |
| AAMP | 0.953 | 0.518 to1.754 | 0.878 | 0.940 | 0.475 to 1.862 | 0.860 |
| ADAM17 | 1.199 | 0.523 to 2.746 | 0.669 | 1.256 | 0.465 to 3.391 | 0.653 |
| ADM2 | 0.928 | 0.484 to 1.778 | 0.822 | 0.912 | 0.454 to1.832 | 0.795 |
| GRK3 | 0.496 | 0.129 to 1.915 | 0.309 | 0.529 | 0.129 to 2.162 | 0.375 |
| AGPAT3 | 0.984 | 0.495 to 1.957 | 0.963 | 0.944 | 0.464 to 1.922 | 0.875 |

qRT-PCR were normalized toβ-actin.

P<0.05 were indicated as bold type

C-index of these genes in FUSCC Cohort is 0.53
